# Supplementary material for: Changes in mitochondrial distribution occur at the axon initial segment in association with neurodegeneration in Drosophila
Source: Biol Open. 2024 Jul 11;13(7):bio060335. doi: 10.1242/bio.060335 (PMC11261633; doi:10.1242/bio.060335)
Supplement: Supplementary information [file biolopen-13-060335-s1.pdf]

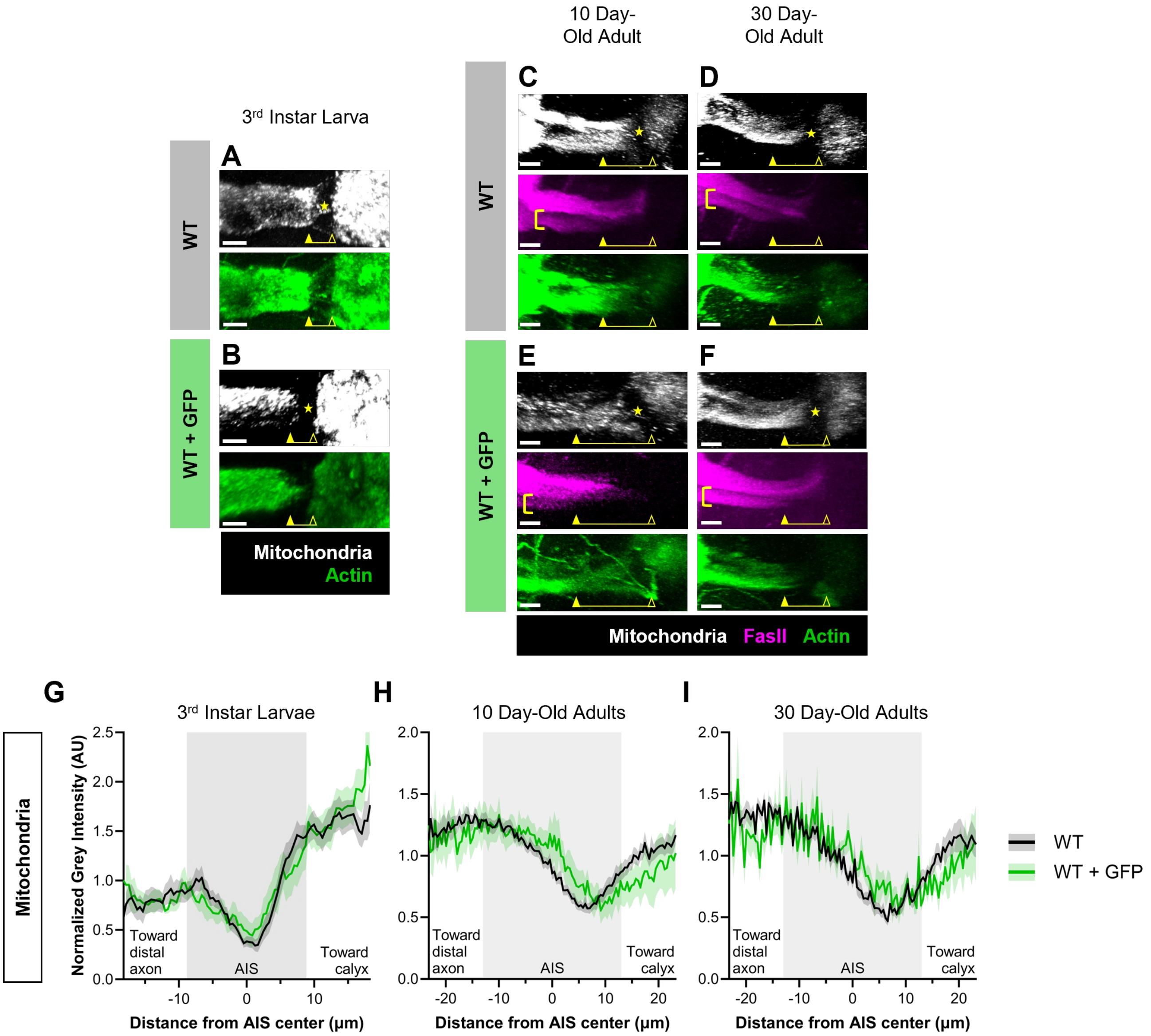

Fig.S1.Expression of an unrelated protein (*UAS-eGFP*) does not alter the partial exclusion of mitochondria from the AIS region of WT MB neurons.

A-B) Representative normalized projections of mitochondrial and actin fluorescence intensity in MBs of WT 3rd instar larvae (A) and WT + GFP 3rd instar larvae (B). Yellow closed arrowheads indicate the distal border of the AIS and yellow open arrowheads indicate the proximal border of the AIS as determined by the pattern of actin distribution as well as morphological criteria (the emergence of bundled axons from the MB calyx). Yellow stars indicate the region of mitochondrial exclusion. Yellow bars represent the length and position of the AIS. White scale bars in the lower left corner of images represents 10 µm.

Biology Open • Supplementary information

C-F) Representative normalized projections of mitochondrial, FasII, and actin fluorescence intensity in MBs of WT 10 day-old adults (C), WT 30 day-old adults (D), WT + GFP 10 day-old adults (E), and WT + GFP 30 day-old adults (F). Yellow closed arrowheads indicate the distal border of the AIS and yellow open arrowheads indicate the proximal border of the AIS as determined by the pattern of actin and FasII distribution as well as morphological criteria. Yellow brackets highlight the MB γ-neurons in which FasII demarcates the distal border of the AIS. Yellow stars indicate the region of maximal mitochondrial exclusion. Yellow bars represent the length and position of the AIS. White scale bars in the lower left corner of images represents 10 µm.

G-I) Quantification of mitochondrial fluorescence intensity across the AIS region of MB neurons of WT and WT + GFP 3rd instar larvae (G), 10 day-old adults (H), and 30 day-old adults (I). Colored lines (black = WT; green = WT + GFP) represent average normalized mitochondrial intensity value while colored shaded regions represent the standard error of the mean (SEM). Note that WT control data is the same pooled data as presented in Figures 3 and 5. The shaded background of the graph represents the average AIS size for larvae or adult, respectively, as determined by Trunova et al., 2011. More negative x-axis values are oriented towards the MB distal axon while more positive x-axis values are oriented towards the MB calyx. N = 12 (WT 3rd instar larvae), 7 (WT + GFP 3rd instar larvae), 24 (WT 10 day-old adults), 7 (WT + GFP 10 day-old adults), 14 (WT 30 day-old adults), and 7 (WT + GFP 30 day-old adults).

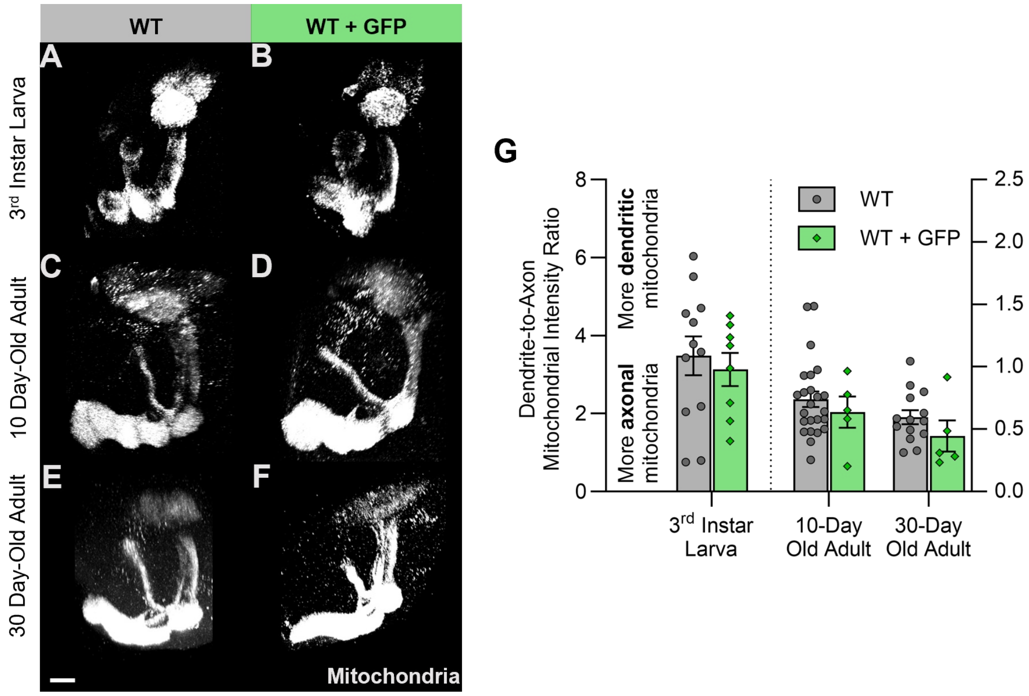

Fig. S2. Expression of an unrelated protein (*UAS-eGFP*) does not alter the ratio of somatodendritic to axonal mitochondria in larval and adult MB neurons.

A-F) Representative normalized projections of mitochondrial fluorescence intensity in the MBs of WT 3rd instar larvae (A), WT + GFP 3rd instar larvae (B), WT 10 day-old adults (C), WT + GFP 10 day-old adults (D), WT 30 day-old adults (E), and WT + GFP 30 day-old adults (F). Scale bar in lower left corner of the figure pane represents 20 µm.

G) Quantification of the ratio of mitochondrial fluorescence intensity in the somatodendritic region to that of the axonal region of MB neurons of WT and WT + GFP 3rd instar larvae, 10 day-old adults, and 30 day-old adults. Note that WT control data is the same pooled data as presented in Figures 1 and 7. Larval groups compared using Mann-Whitney *U* test (Kolmogorov-Smirnov normality test, $p < .05$) and adult groups compared using two-way ANOVA with Šidák's multiple comparison test. *p* values: * < .05; ** < .01, *** < .001, **** < .0001. N = 12 (WT 3rd instar larvae), 8 (WT + GFP 3rd instar larvae), 24 (WT 10 day-old adults), 5 (WT + GFP 10 day-old adults), 14 (WT 30 day-old adults), and 5 (WT + GFP 30 day-old adults).

Table S1.

Available for download at
https://journals.biologists.com/bio/article-lookup/doi/10.1242/bio.060335#supplementary-data

Biology Open • Supplementary information
